# Genetic background modifies CNS‐mediated sensorimotor decline in the AD‐BXD mouse model of genetic diversity in Alzheimer's disease

**DOI:** 10.1111/gbb.12603

**Published:** 2019-08-19

**Authors:** Kristen M. S. O'Connell, Andrew R. Ouellette, Sarah M. Neuner, Amy R. Dunn, Catherine C. Kaczorowski

**Affiliations:** ^1^ The Jackson Laboratory Bar Harbor Maine; ^2^ Department of Anatomy and Neurobiology The University of Tennessee Health Science Center Memphis Tennessee

**Keywords:** alzheimer's disease, balance, coordination, dementia, genetics, muscle, noncognitive, preclinical AD, sensorimotor function

## Abstract

Many patients with Alzheimer's dementia (AD) also exhibit noncognitive symptoms such as sensorimotor deficits, which can precede the hallmark cognitive deficits and significantly impact daily activities and an individual's ability to live independently. However, the mechanisms underlying sensorimotor dysfunction in AD and their relationship with cognitive decline remains poorly understood, due in part to a lack of translationally relevant animal models. To address this, we recently developed a novel model of genetic diversity in Alzheimer's disease, the AD‐BXD genetic reference panel. In this study, we investigated sensorimotor deficits in the AD‐BXDs and the relationship to cognitive decline in these mice. We found that age‐ and AD‐related declines in coordination, balance and vestibular function vary significantly across the panel, indicating genetic background strongly influences the expressivity of the familial AD mutations used in the AD‐BXD panel and their impact on motor function. Although young males and females perform comparably regardless of genotype on narrow beam and inclined screen tasks, there were significant sex differences in aging‐ and AD‐related decline, with females exhibiting worse decline than males of the same age and transgene status. Finally, we found that AD motor decline is not correlated with cognitive decline, suggesting that sensorimotor deficits in AD may occur through distinct mechanisms. Overall, our results suggest that AD‐related sensorimotor decline is strongly dependent on background genetics and is independent of dementia and cognitive deficits, suggesting that effective therapeutics for the entire spectrum of AD symptoms will likely require interventions targeting each distinct domain involved in the disease.

## Graphical abstract

Sensorimotor deficits in Alzheimer's disease are distinct from AD‐related cognitive decline or normal age‐related decline.

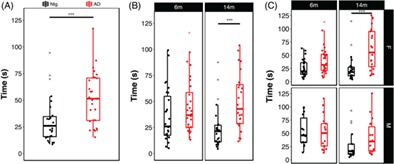

## 1 INTRODUCTION

Alzheimer's dementia (AD) is defined by the slow progression of cognitive deficits, including memory loss and dementia, accompanied by the accumulation of β‐amyloid (Aβ) plaques and hyperphosphorylated tau tangles.^1^ However, patients with AD often experience additional noncognitive symptoms that significantly impact daily life for both patients and caregivers and lead to an inability to live independently, requiring long‐term care.^2^, ^3^, ^4^, ^5^, ^6^ Among these noncognitive symptoms are deficits in sensorimotor function such as gait slowing, loss of balance and coordination, sarcopenia and muscle weakness and increased frailty. Furthermore, the emergence of these symptoms during the preclinical stage of AD is associated with worse AD‐related cognitive decline than in individuals who do not exhibit motor‐related symptoms.^4^

Development of motor deficits prior to the hallmark memory loss associated with AD^2^, ^3^ suggests that they may represent some of the very earliest events in the pathogenesis of AD. Unfortunately, motor dysfunction and other noncognitive symptoms in AD are poorly understood compared with the cognitive deficits and memory loss so closely associated with the disease. It therefore remains unknown whether motor impairment represents an early biomarker of disease, or if it is part of a chain of causal events leading to dementia. It is essential to determine whether AD‐related decline in cognitive and motor function share a common mechanism. If shared pathogenesis exists Understanding the causal factors underlying motor symptoms may enhance our ability to identify new pathways for therapeutics that address both domains. Alternatively, if they have distinct causal mechanisms, then therapies targeting cognitive symptoms may be ineffective in treating motor dysfunction and thus, fail to prevent or delay the loss or independence or the need for long‐term institutional care.

Significant progress in understanding the pathophysiology of AD has been made using mouse models incorporating familial mutations in amyloid precursor protein (*APP*) and/or presenilin‐1 (*PSEN1*), originally designed to recapitulate the cognitive symptoms of AD. Notably, several of these models have been reported to also exhibit sensorimotor deficits, suggesting that motor impairments are an inherent part of the disease process.^7^, ^8^, ^9^, ^10^, ^11^, ^12^, ^13^ However, infrequent assessment of motor phenotypes in AD animal models, variability in the tests used to assess motor function, and use of a single sex has led to conflicting reports on the impact of AD transgenes on motor function.^7^, ^10^, ^11^, ^14^, ^15^

The etiology of AD in humans is complex and although age is the greatest risk factor for developing AD, it is increasingly clear that genetics and family history play a significant role.^16^, ^17^ Most animal models are developed on single or a few inbred backgrounds,^18^ with little or no genetic variation, presenting a challenge for identifying and investigating AD symptoms such as motor dysfunction, which can vary considerably in its presentation in human populations. As such, a major barrier to understanding the overlap (or lack thereof) of mechanisms underlying motor dysfunction and cognitive symptoms in AD is a paucity of translationally relevant animal models that recapitulate individual differences in symptom onset and progression in the human population.

To address this, we recently developed the AD‐BXD panel,^19^ which combines the well‐characterized 5XFAD model of AD^20^ with the BXD genetic reference panel^21^, ^22^ to create a novel AD model that incorporates both causal AD mutations and naturally‐occurring genetic diversity to better model the human disease. In the present study, we assess the impact of AD, normal aging and naturally occurring genetic variation on sensorimotor‐related phenotypes and their relationship to cognitive outcomes using the AD‐BXD panel. Because the panel also includes the nontransgenic controls, we can distinguish the influence of familial AD mutations from the normal decline in motor skills commonly observed in aging. We hypothesized that the AD‐BXD panel would exhibit age‐related decline in sensorimotor function that is exacerbated by the presence of the AD transgene and that diverse genetic backgrounds would influence the expressivity of the 5XFAD transgene to modify the onset and severity of motor‐related phenotypes.

## 2 METHODS

### 2.1 Mice

Generation of the AD‐BXD panel was described in detail in References.^19^ Briefly, female congenic C57Bl/6J mice hemizygous for the 5XFAD transgene^20^ (MMRRC Stock No: 34848‐JAX) were crossed to male mice from strains selected from the BXD genetic reference panel.^21^ The resulting F1 offspring are isogenic recombinant inbred mice carrying one maternally derived *B* allele and one paternally derived *B* or *D* allele at each genomic locus. Furthermore, ~50% of F1 mice carry the 5XFAD transgene (termed AD‐BXD), while the other ~50% are nontransgenic littermate controls (termed Ntg‐BXDs). Mice from a total of 27 Ntg‐ and AD‐BXD (27 female, 18 male) strains were used in this study; mice were genotyped for the 5XFAD transgene by either the Transgenic Genotyping Service at The Jackson Laboratory or Transnetyx, Inc. All mice were fed a standard laboratory mouse chow (Teklad 8604) and both food and water were available ad libitum. All mice were kept on a 12:12 light cycle and were phenotyped as previously described.^19^

All experiments involving mice were performed at the University of Tennessee Health Science Center and were approved by the Institutional Care and Use Committee at that institution and carried out in accordance with the standards of the Association for the Assessment and Accreditation of Laboratory Animal Care (AAALAC) and the NIH Guide for the Care and Use of Laboratory Animals.

### 2.2 Sensorimotor phenotyping

Sensorimotor function was assessed at 6 and 14 months of age in the Ntg‐BXD and AD‐BXD panels. A total of 894 mice (575 female, 319 male) across a total of 27 AD‐ and Ntg‐BXD strains were included in this study. The sensorimotor tasks used were narrow beam, negative geotaxis (incline screen) and grip strength.^19^ *Narrow beam*: Briefly, the narrow beam task was used to assess motor and balance coordination by placing each mouse in the center of a 1 m long beam 12 mm wide that was elevated 50 cm above a table surface (Maze Engineers, Boston, Massachusetts). The time taken in seconds for each mouse to cross the beam to a safe platform on either side was recorded. A maximum time limit of 180 seconds was imposed; if a mouse fell from the beam, the maximum time of 180 seconds was given. The average of three trials for the task was used to assess the performance for each mouse and the average used for statistical analysis.

*Incline screen*: The incline screen task was used to assess vestibular and/or proprioceptive function. Each mouse was placed nose‐down in the center of a wire mesh grid (1 × 1 cm) positioned at a 45° angle (Harvard Apparatus). The time taken for a mouse to reorient itself with its nose facing upwards (negative geotaxis) was recorded; the average of three trials for each mouse was used for analysis. As with narrow beam, a 180 seconds maximum time limit for righting was used.

*Grip strength*: Muscle strength was measured using a standard grip strength meter. Each mouse was placed horizontally on the wire grid of the apparatus (Columbus Instruments) with all four paws grasping the grid and then gently pulled away from the grid by the base of its tail until its grip released to measure the force exerted. The average of three trials for each mouse was recorded and used for analysis.

### 2.3 Data analysis and availability

All data and statistical analysis were performed using R (version 3.5.3). A univariate ANOVA was used for each sensorimotor task using genotype, age, sex and strain as fixed factors. Data are reported as mean ± SE of the mean in both the main text and figure legends. A two‐sided Pearson's correlation test was used to assess the relationship of decline between the Ntg‐BXD and AD‐BXD on these tasks. CFA and CFM measures used for correlations in Figures 3 and 5 are from.^19^ All raw data used in this study are available through the Synapse AMP‐AD Knowledge Portal (https://www.synapse.org/#!Synapse:syn17016211). Strain averaged behavioral data has also been deposited at http://genenetwork.org. Heritability estimates for the sensorimotor phenotypic traits in the AD‐ and Ntg‐BXDs were determined by calculating the ratio of between‐strain variance to total sample variance (variance due to both genetic and technical/environmental factors) according to.^23^

## 3 RESULTS

### 3.1 Balance and motor coordination are impaired in AD‐BXD mice in a genetic‐background dependent manner

Since balance and coordination skills are often impaired in human AD patients,^3^ we assessed these sensorimotor domains in the AD‐BXD panel using the narrow beam task, which is a well‐characterized and validated assay for balance and coordination in mice.^24^, ^25^ To determine the impact of genetic background on AD‐ and age‐related impairments in these domains, we measured performance on the narrow beam apparatus in 27 strains of AD‐BXD mice (27 strains female, 18 strains male) at 6 and 14 months (m) of age. We also tested age‐matched Ntg‐BXD strains to assess the impact of normal aging on this task.

**Figure 1:**
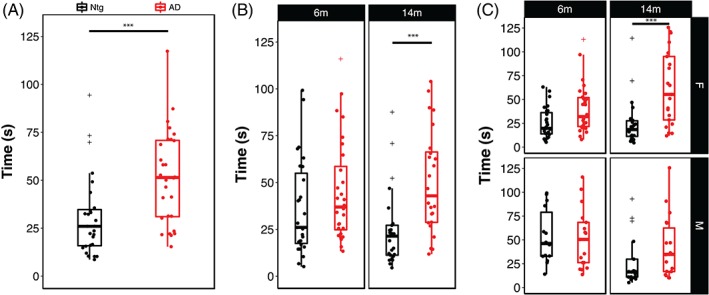
Presence of the high‐risk AD transgene impairs motor coordination and balance in an age‐ and sex‐dependent manner. (A) Mice carrying the 5XFAD transgene take significantly longer to cross the narrow beam apparatus, indicating impaired balance and coordination. (B) Stratification of narrow beam performance by age and (C) by age and sex. Each point represents a strain average and statistical outliers are indicated by a + sign. *** = adj. *P* < .0001 following post hoc testing using Tukey's HSD

As shown in Figure 1A, as a population, AD‐BXD mice took significantly longer to cross the beam compared with the non‐carrier controls (Genotype: F_1,886_ = 24.5, *P* < .0001). There was a main effect of age on narrow beam performance (6 m v 14 m: F_1, 886_ = 3.9, *P* < .05) and presence of the AD transgene significantly exacerbated age‐related decline (Age*Genotype: F_1,886_ = 7.6, *P* < .005) (Figure 1B). Post hoc comparisons using the Tukey HSD test indicated that this effect was primarily driven by the presence of the AD transgene and was not due to decline due to normal aging, as there was no significant effect of age in the Ntg‐BXD (6 m‐14 m, adj. *P* > .1). Furthermore, although there was a trend for 6 m AD‐BXD mice to perform worse on this task than their age‐matched Ntg‐BXD controls, this difference was not significant (adj. *P* > .1). Lastly, although there was no significant main effect of sex on narrow beam performance across the panel (Sex: F_(1, 886)_ < 1, n.s.), there were significant interactions between both age and sex (Age*Sex: F_(1, 886)_ = 9.16, *P* < .005) and sex and genotype (Sex*Genotype: F_(1, 886)_ = 7.9, *P* < .005), with post hoc comparisons suggesting that impairment due to age and AD status was greater in females than males (Figure 1C).

**Figure 2:**
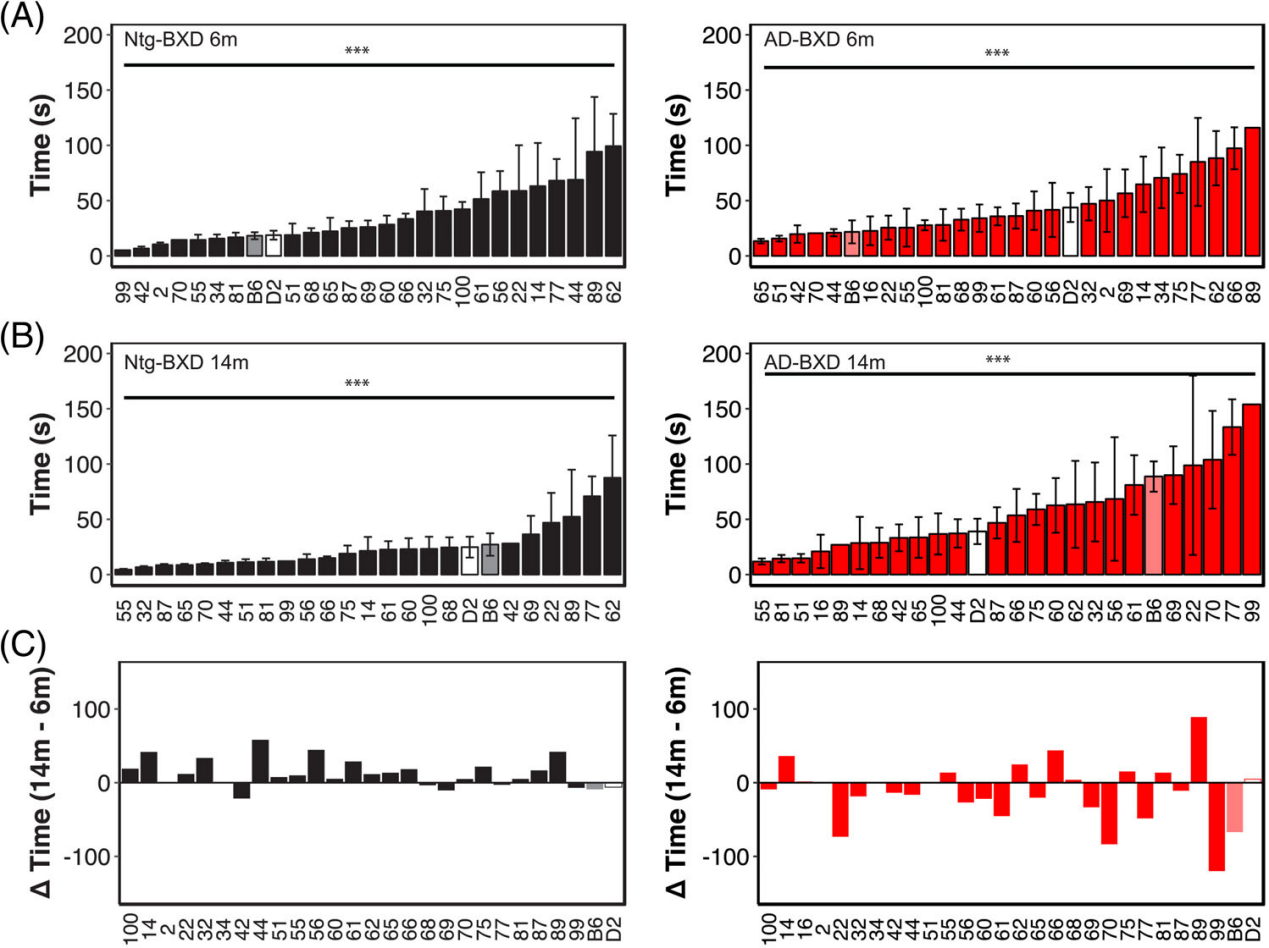
Genetic background influences motor coordination and balance on the narrow beam task in both normal aging and AD. (A) Mean time to cross the narrow beam averaged by strain in 6 m old Ntg‐BXD (*left*) and AD‐BXD (*right*) strains. (B) Mean time to cross the narrow beam averaged by strain in 14 m old Ntg‐BXD (*left*) and AD‐BXD (*right*) strains. (C) Average age‐related decline by strain in the Ntg‐BXD (*left*) and AD‐BXD (*right*) panels. Decline was calculated by subtracting performance at 6 m of age from that measured at 14 m of age. Data are presented as mean ± SEM. In all panels, number on the x‐axis indicates the BXD strain used to generate each line. *** = *P* < .0001

**Figure 3:**
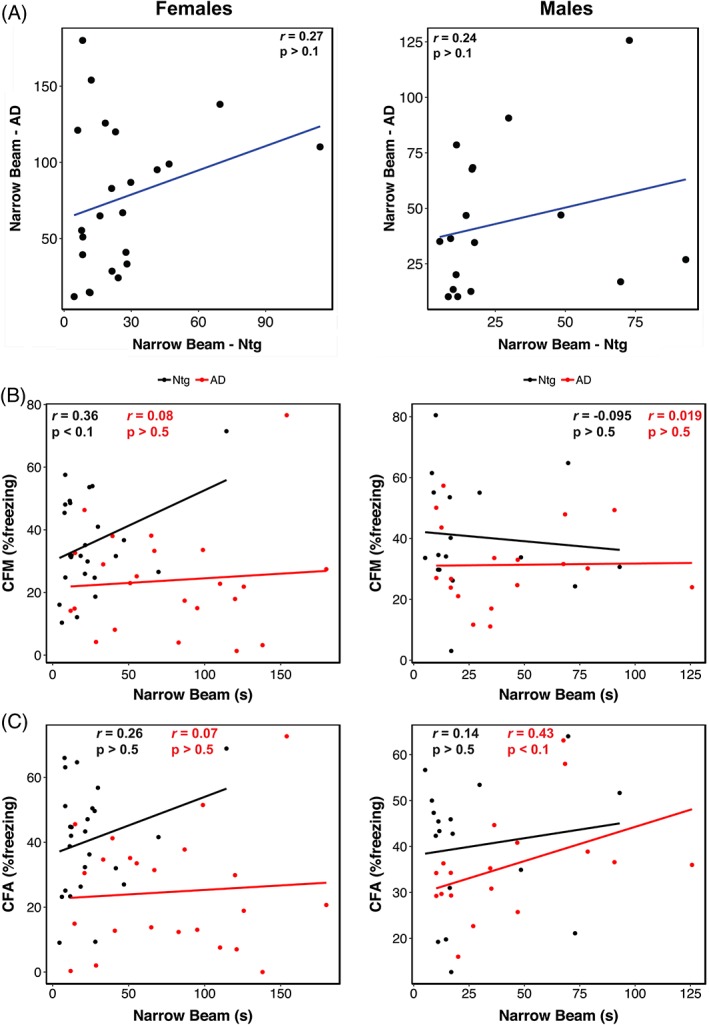
Lack of trait correlations suggests AD‐related decline in narrow beam performance has distinct genetic mechanisms from normal aging or AD‐related cognitive phenotypes. (A) Scatterplot of narrow beam performance at 14 m of age in female (*left*) and male (*right*) Ntg‐ and AD‐BXD strains. Narrow beam performance in 14 m old female (*left*) and male (*right*) plotted against contextual fear acquisition (CFA) (A) and contextual fear memory (CFM) (B). For all panels, each point represents a strain mean. Black = Ntg‐BXD; red = AD‐BXD

To assess the influence of genetic background on balance and coordination, we compared narrow beam performance across the Ntg‐ and AD‐BXD strains phenotyped in this study. As shown in Figure 2, genetic background plays a significant role in narrow beam performance as evidenced by a significant main effect of background strain in both the Ntg‐ (black bars) and AD‐BXD strains (red bars) (Strain_Ntg_: F_(26, 363)_ = 3.35, *P* < .0001_;_ Strain_AD_: F_27,474_) = 2.7, *P* < .0001) and a high degree of heritability for this task (Table 1). As expected from our population‐level analyses, although there is a significant main effect of strain at both 6 and 14 m in the Ntg‐BXDs (Figure 2A,B, *left panels*), there is no effect of age, with most Ntg‐BXD strains performing as well or better at 14 m compared with 6 m (Figure 2C). On the other hand, both age and background strain were significant in the AD‐BXD population (Figure 2, *right panels*), suggesting that age related decline is exacerbated in most (but not all) AD‐BXD strains. Notably, although there are significant main effects of both strain and genotype as described above, there is no significant correlation between the Ntg‐ and AD‐BXD strains on narrow beam performance in either males (*r* = .24, *P* > .1, *df* = 15) or females (*r* = .27, *P* > .1, *df* = 21) (Figure 3A), suggesting that genotype*strain interactions have a greater impact on narrow beam performance than strain alone. Consistent with the pronounced influence of background strain, heritability ($h_{\mathrm{RI}\bar{x}}^{2}$) estimates comparing the between‐strain variance (an estimate of variation due to genetic factors) to total sample variance (an estimate of variation due to environmental, technical and genetic factors) indicate that genetic factors significantly influence phenotypic variation (Table 1).

**Table 1:** Heritability estimates for sensorimotor traits in Ntg‐ and AD‐BXD strains

Ntg‐BXD				
Trait	Between‐strain variability	Average within‐strain variability	Average n/strain	$h_{\mathrm{RI}\bar{x}}^{2}$
6 m narrow beam	667.3893	2351.59	8.68	0.7
14 m narrow beam	414.7125	1428.3	7.39	0.7
6 m inclined screen	63.05047	147.417	8.68	0.8
14 m inclined screen	92.87501	425.05	7.39	0.6
6 m grip strength	0.08793	0.188	8.68	0.8
14 m grip strength	0.074671	0.179	7.39	0.8

AD‐BXD				
Trait	Between‐strain variability	Average within‐strain variability	Average n/strain	$h_{\mathrm{RI}\bar{x}}^{2}$
6 m narrow beam	734.1014	2697.44	10.77	0.7
14 m narrow beam	1357.38	4261.44	9.125	0.7
6 m inclined screen	90.23455	361.28	10.77	0.7
14 m inclined screen	307.7143	1362.3	9.125	0.7
6 m grip strength	0.135841	0.194	10.77	0.9
14 m grip strength	0.075719	0.211	9.125	0.8

*Note*: Heritability ($h_{\mathrm{RI}\bar{x}}^{2}$) was calculated as the ratio of between‐strain variance (ie, trait variance due to genetic factors) to total variance (ie, variance due to technical and environmental factors, assessed as within‐strain variance, plus genetic variance) normalized to the average number of biological replicates per strain (n) according to.^23^

The AD‐BXD panel exhibits significant strain‐dependent cognitive decline,^19^ so to determine whether cognitive performance in this panel correlates with sensorimotor ability, we assessed the relationship between narrow beam performance and memory as measured using the contextual fear memory task.^19^ We found no significant correlation between motor performance and either short‐term memory (CFA, contextual fear acquisition) or long‐term memory (contextual fear memory, CFM; from Reference^19^) in either Ntg‐ or AD‐BXD mice of either sex (Figure 3B,C), suggesting that the mechanism(s) underliying deficits in balance and coordination are, in part, unrelated to aging or AD‐related cognitive impairment in this panel of mice.

### 3.2 AD‐related impairments in motor coordination and vestibular function

**Figure 4:**
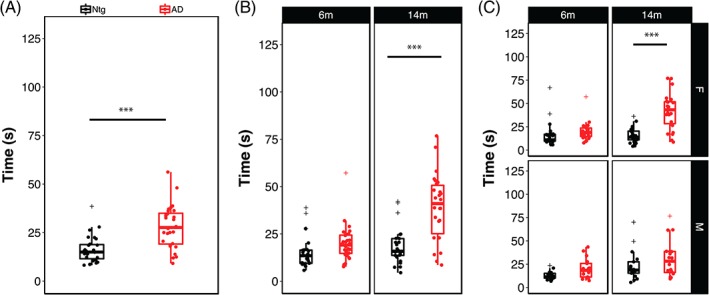
Impairments in motor coordination, vestibular function and proprioception are exacerbated by the AD transgene. (A) Mice carrying the 5XFAD transgene take significantly longer to right themselves on the inclined screen apparatus, indicating impaired proprioception and vestibular function. (B) Stratification of inclined screen performance by age and (C) by age and sex. Each point represents a strain average and statistical outliers are indicated by a + sign. *** = adj. *P* < .0001 following post hoc testing using Tukey's HSD

Human AD patients often exhibit impairments in balance and orientation suggestive of deficits in vestibular function and proprioception, domains that are assessed in mice using the inclined screen test. In this test, mice are placed head down on a wire mesh screen fixed at a 45° angle and their natural reflex to reposition themselves head up measured, with longer righting times suggestive of impairments in proprioceptive and vestibular systems.^26^ There was a significant effect of AD genotype on this task (Figure 4A), with AD‐BXD mice requiring significantly longer time to reorient themselves than Ntg‐BXD mice (Genotype: F_[1886]_ = 55.21, *P* < .0001). There was also a significant effect of age on inclined screen performance (Figure 4B), with 14 m old mice taking significantly longer than 6 m old mice (Age: F_(1,886)_ = 59.8, *P* < .0001). For this task, there was a significant interaction between age and genotype (Age*Genotype: F_(1,886)_ = 20.33, *P* < .0001). As with the narrow beam task, post hoc multiple comparisons indicated that Ntg‐BXD mice do not exhibit significant age‐related decline (adj. *P* > .1), while inclined screen performance is significantly worse with age in the AD‐BXD mice (adj. *P* < .000001), suggesting a strong interaction between age and genotype on vestibular function and proprioception. Again, there was no main effect of sex on inclined screen performance (Sex: F_(1,886)_ = 1.5, *P* > .1), nor was there a significant interaction between sex and age (Age*Sex: F_(1,886)_ = 1.14, *P* > .1). There was an interaction between sex and genotype (Genotype*Sex: F_(1,886)_ = 3.87, *P* < .05), which post hoc comparisons indicate is due to worse performance by AD mice relative to their Ntg counterparts, with females affected more than males (Male adj. *P* < .005; female adj. *P* < .000001, Figure 4C).

**Figure 5:**
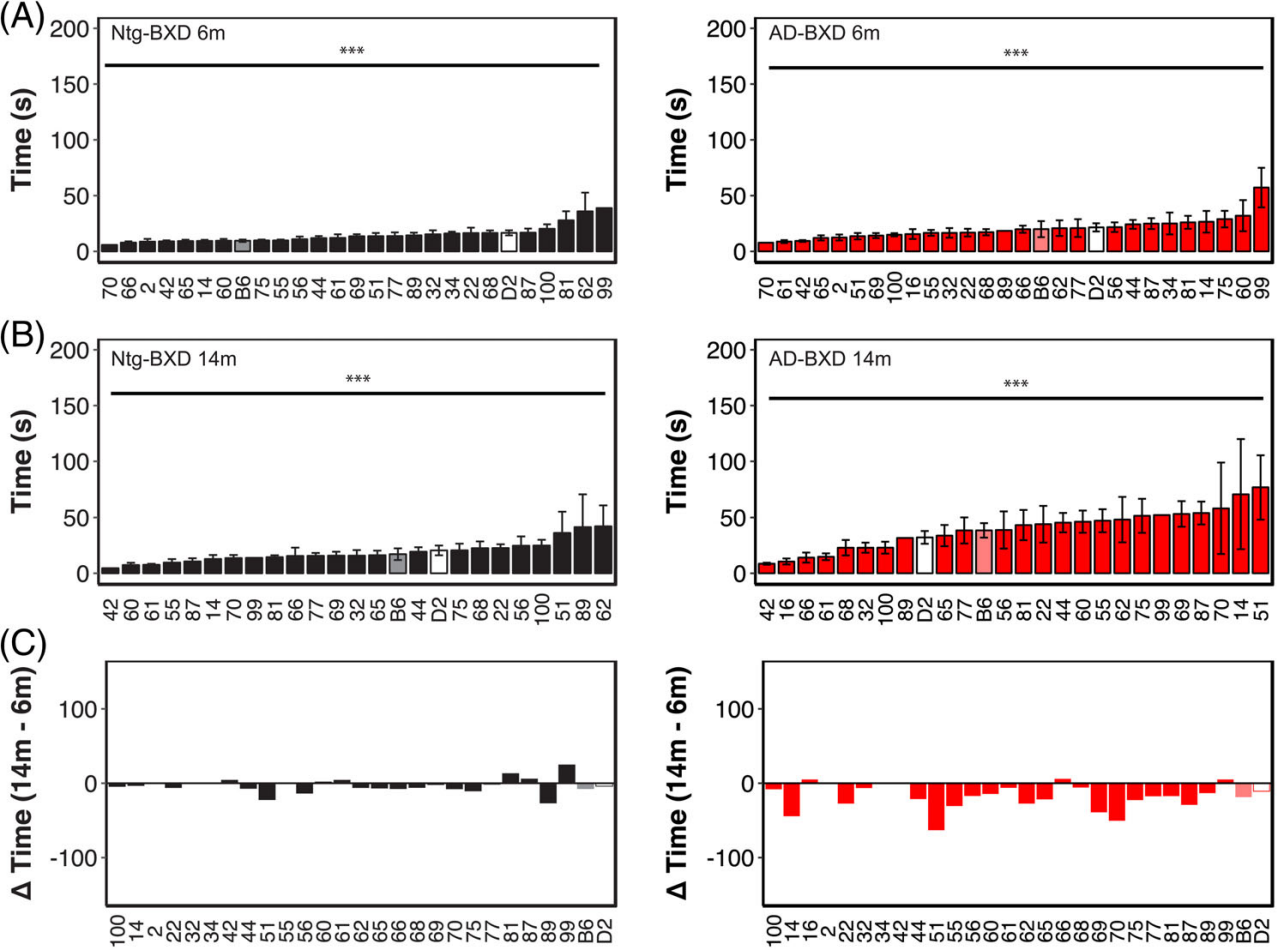
Genetic background influences vestibular function and proprioception on the inclined screen task in both normal aging and AD. (A) Mean righting time on the inclined screen averaged by strain in 6 m old Ntg‐BXD (*left*) and AD‐BXD (*right*) strains. (B) Mean righting time on the inclined screen average by strain in 14 m old Ntg‐BXD and AD‐BXD strains. (C) Average age‐related decline by strain in the Ntg‐BXD (*left*) and AD‐BXD (*right*) panels. Decline was calculated by subtracting performance at 6 m of age from that measured at 14 m of age. Data are presented as mean ± SEM. In all panels, number on the x‐axis indicates the BXD strain used to generate each line. *** = *P* < .0001

**Figure 6:**
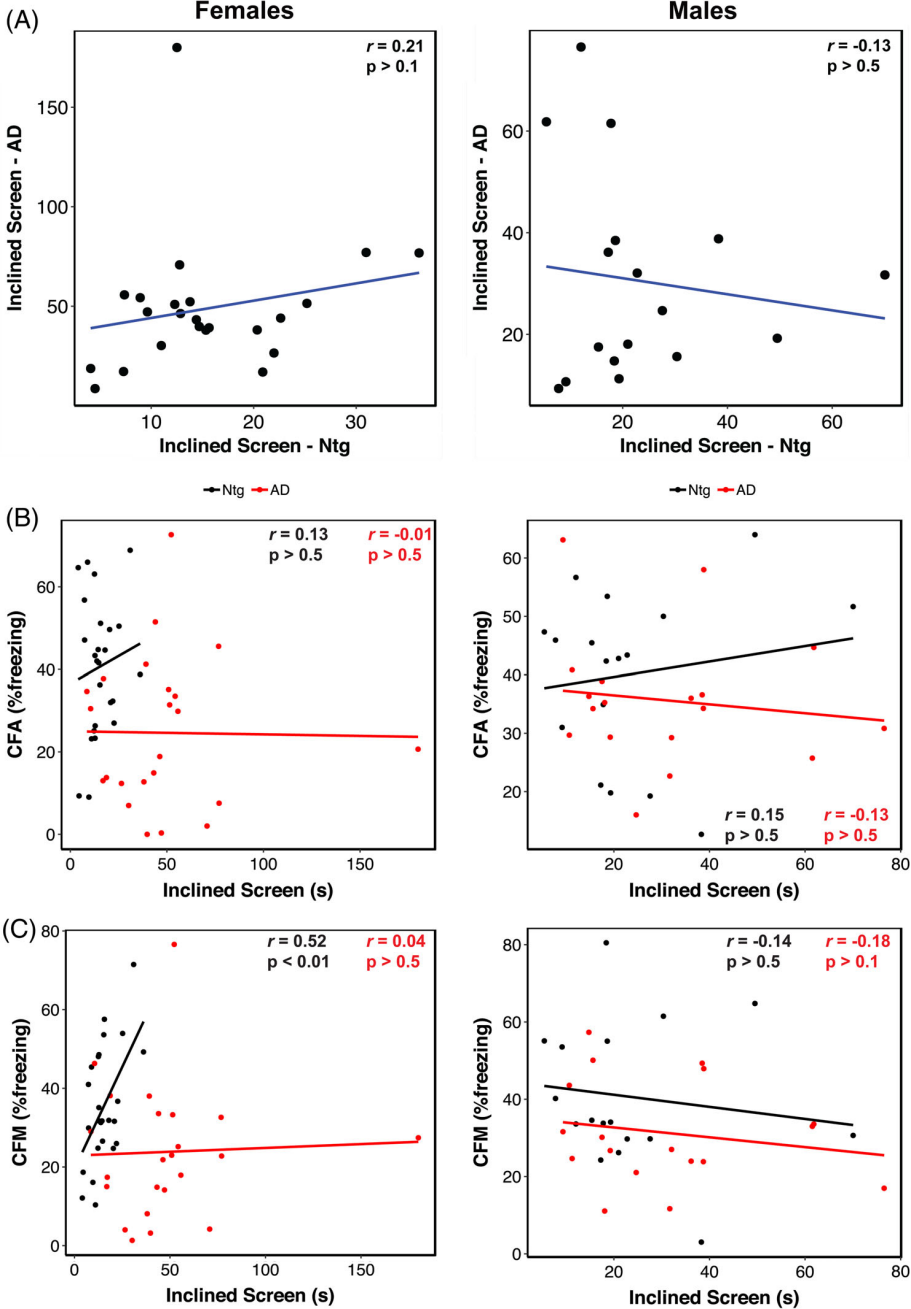
Lack of trait correlations indicates AD‐related declines in righting on the inclined screen are controlled by distinct genetic mechanisms from either normal aging or AD‐related cognitive phenotypes. (A) Scatterplot of inclined screen performance at 14 m of age in female (*left*) and male (*right*) Ntg‐ and AD‐BXD strains. Inclined screen performance in 14 m old female (*left*) and male (*right*) plotted against contextual fear acquisition (CFA) (A) and contextual fear memory (CFM) (B). For all panels, each point represents a strain mean. Black = Ntg‐BXD; red = AD‐BXD

As with the narrow beam task, we also assessed the impact of genetic variation on inclined screen performance by examining righting latency as a function of background strain (Figure 5). Analysis of individual strain performance on this task showed a significant main effect of strain in both Ntg‐ and AD‐BXD strains (Strain_Ntg_: F_(26,339)_ = 2.04, *P* < .005; Strain_AD_: F_(27, 474)_ = 2.2, *P* < .0005), once again suggesting a strong influence of naturally occurring genetic variation on vestibular function and proprioception (Figure 5A). On this task, there was a no significant interaction between strain and age in either genotype (Age*Strain_Ntg_: F_(24,339)_ < 1, n.s.; Age*Strain_AD_: F_(25, 450)_ = 1.2, *P* > .1). We found a significant interaction between strain, sex and genotype, indicating that female mice from AD strains are impaired relative to male AD strains, which may account for the slight difference in decline between 6 and 14 m of age shown in Figure 5C. As with narrow beam, there was no significant correlation between inclined screen performance in Ntg‐BXD strains compared with AD‐BXD strains (females: *r* = .21, *P* > .1, *df* = 21; males: *r* = −.13, *P* > .1, *df* = 15) (Figure 6A), suggesting that expressivity of the transgene is not equal across strains, but instead depends on genetic background. As with narrow beam, $h_{\mathrm{RI}\bar{x}}^{2}$ estimates indicate that genetic background accounts for much of the variation in inclined screen performance (Table 1).

Similar to the narrow beam task, we found no correlation between performance on the inclined screen test and long‐term CFM in male Ntg‐ and AD‐BXD strains or female AD‐BXD strains (Figure 6B,C), suggesting impairments in vestibular function and proprioception may be independent of cognitive decline. Interestingly, we did observe a significant positive correlation between inclined screen performance and long‐term CFM in female Ntg‐BXD strains (Figure 6C, *left*); however, it should be noted that there is very little phenotypic variation in the inclined screen task compared with CFM, with all female Ntg‐BXD strains performing very well on this task.

### 3.3 Muscle strength decreases with age in both Ntg‐ and AD‐BXD mice

**Figure 7:**
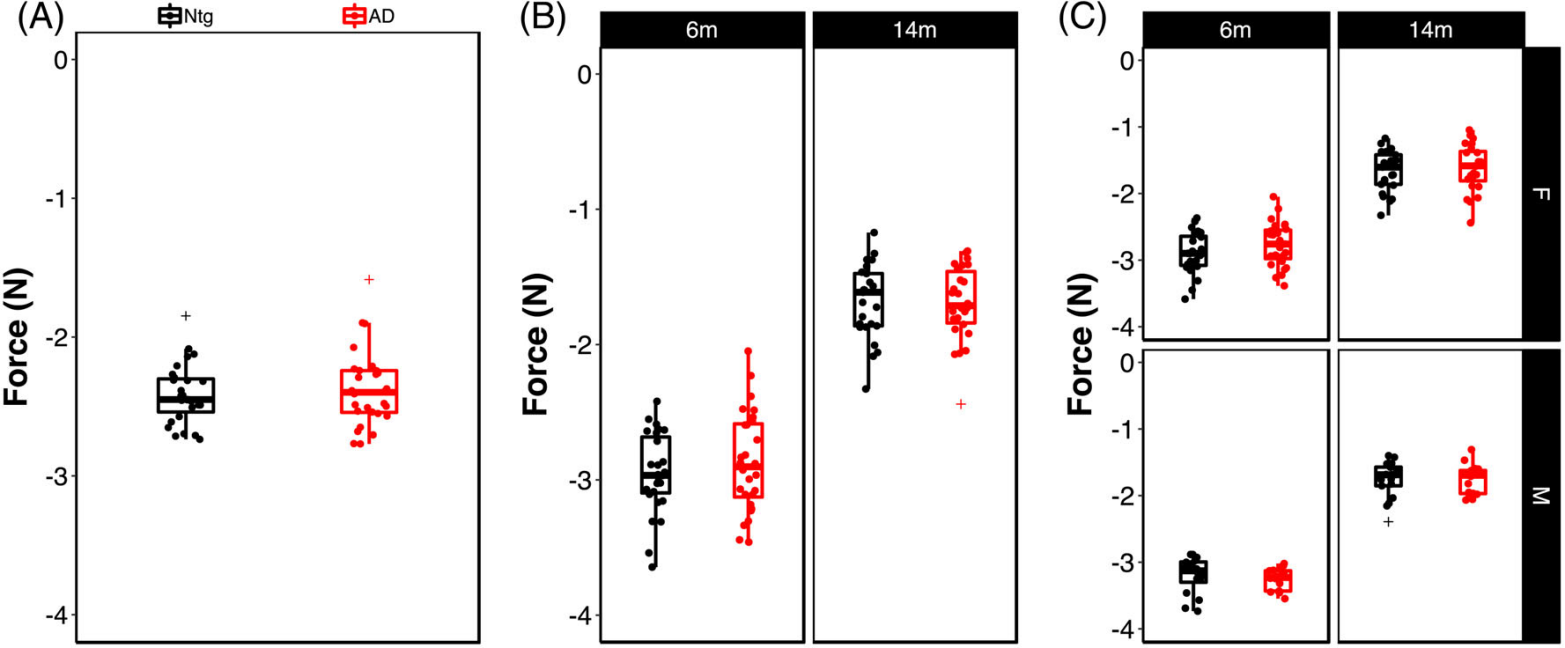
Age, but not sex or genotype, is associated with loss of grip strength. (A) Grip strength in all four paws in Ntg‐ and AD‐BXD strains. (B) Grip strength stratified by age and (C) by age and sex. Each point represents a strain average and statistical outliers are indicated by a + sign

Sarcopenia and muscle weakness are common in aging humans and grip strength is frequently used to monitor strength in the aging population. As in humans, grip strength can be readily assessed in mice and may show declines in muscle mass and strength in aging mice. To assess grip strength, mice were allowed to grasp the grip strength apparatus with all four paws while being held by the tail. Muscle strength in the fore‐ and hindlimbs was measured by pulling the mouse away from the grid by the tail to determine the force exerted during the animal's attempt to maintain its grip on the bar; greater force is indicated by more negative values. As shown in Figure 7A, there was no significant effect of genotype on grip strength, with Ntg and AD mice performing nearly equally (Genotype: F_(1,892)_ < 1, n.s.). As expected, there was a significant effect of age on grip strength, with both Ntg and AD mice exhibiting significant decline with age from 6 to 14 m (Age: F_(1,892)_ = 1444.70, *P* < .00001) (Figure 7B) and female mice exhibiting lower grip strength than male mice (Sex: F_(1,892)_ = 40.97, *P* < .00001) (Figure 7C). However, there was no significant interaction of genotype with age and/or sex, suggesting that all mice decline similarly with age, regardless of AD carrier status, with female mice exhibiting the expected sexual dimorphism in muscle strength (Figure 7C).

**Figure 8:**
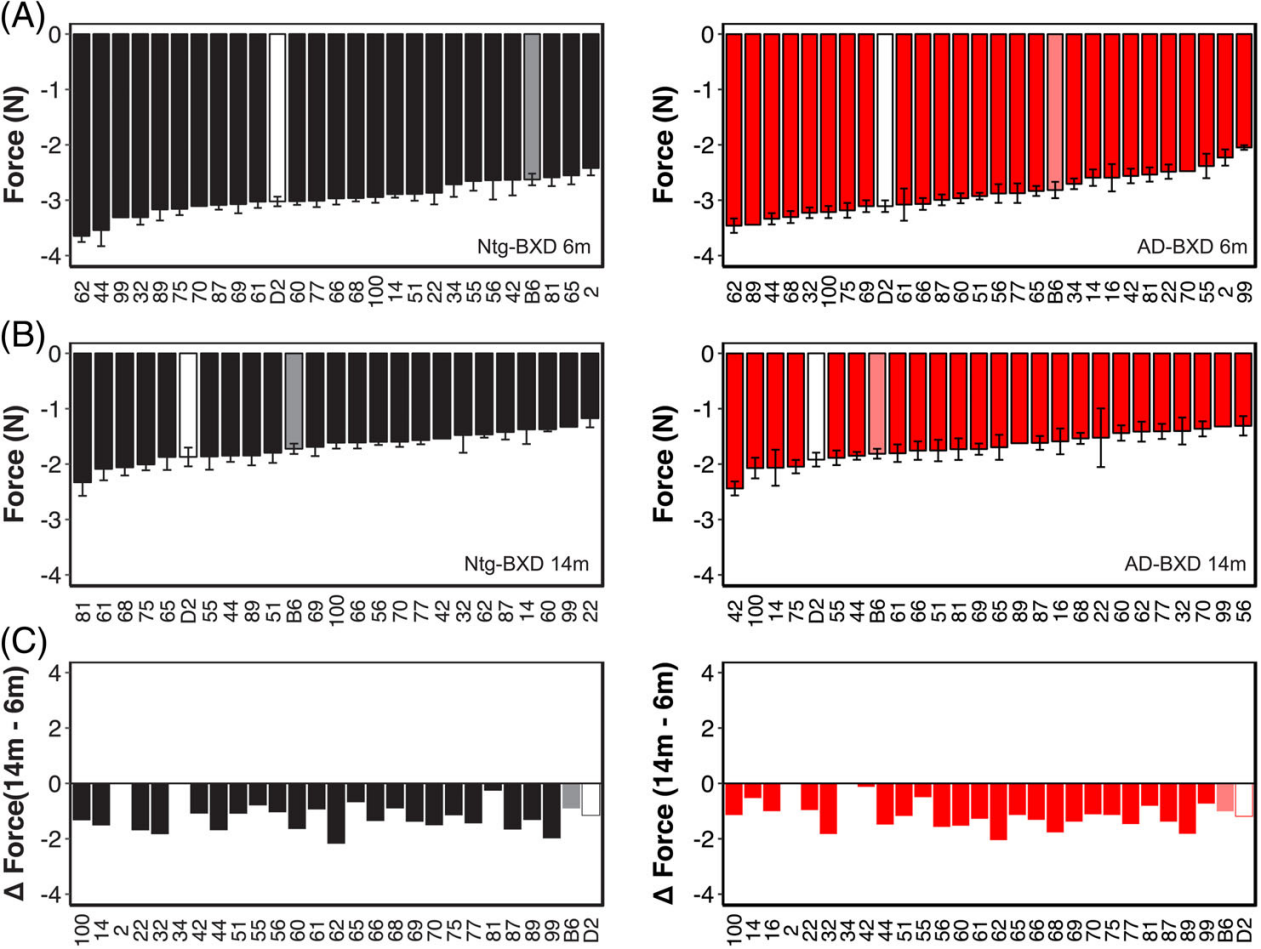
Genetic background influences grip strength in both Ntg‐ and AD‐BXD strains. (A) Mean grip strength in all four paws per strain in 6 m old Ntg‐BXD (*left*) and AD‐BXD (*right*) strains. (B) Mean grip strength in all four paws in 14 m old Ntg‐BXD (*left*) and AD‐BXD (*right*) strains. (C) Average‐age related decline in four‐paw grip strength in the Ntg‐BXD (*left*) and AD‐BXD (*right*) panels. Decline was calculated by subtracting grip strength at 6 m of age from that measured at 14 m of age. Data are presented as mean ± SEM. In all panels, number on the x‐axis indicates the BXD strain used to generate each line

We determined the influence of genetic background on grip strength by assessing average grip strength for each strain at 6 and 14 m (Figure 8A). As shown in Figure 6A,B, there is significant strain‐dependent variation in grip strength in both Ntg and AD populations (Strain_Ntg_: F_(26, 363)_ = 3.02, *P* < .00001; Strain_AD_: F_(__27,474__)_ = 4.2, *P* < .00001) and a significant interaction between age and strain (Age*Strain_Ntg_: F_(24,363)_ = 2.6, *P*< .00001; Age*Strain_AD_: F_(25,449)_ = 2.34, *P* < .0005). Consistent with our finding that there was no influence of genotype on grip strength, we found no significant interaction of genotype with strain (F_(26,839)_ < 1, n.s.). Taken together, these results suggest that age and genetic background profoundly impact muscle strength and decline and that this is not impacted by the presence of the AD transgene. As with the other sensorimotor traits assessed in this study, the correlation between grip strength in the Ntg‐BXD and AD‐BXD is not significant, although it is stronger than observed for narrow beam and inclined screen (*r* = .35, *P* > .1, *df* = 21), suggesting that individual strains may perform more similarly at this task.

## 4 DISCUSSION

### 4.1 AD‐BXD panel recapitulates the exacerbated age‐related sensorimotor deficits associated with AD

Although dementia and cognitive decline are the hallmark diagnostic symptoms of AD in humans, many AD patients also experience impairments in noncognitive domains, including phenotypes associated with sensorimotor function such as coordination, balance, muscle strength and proprioception.^2^, ^3^, ^4^, ^6^, ^27^, ^28^, ^29^ In this study, we investigated the impact of naturally occurring genetic variation on age‐ and AD‐related sensorimotor decline using our recently developed AD‐BXD genetic reference panel.^19^ There was significant variation in balance, coordination and vestibular function—both Ntg‐ and AD‐BXD mice showed age‐related declines in these phenotypes and the presence of familial AD mutations in the AD‐BXD mice exacerbated this decline. There was a significant effect of sex on AD‐related motor decline, with female AD‐BXD mice exhibiting greater impairment on motor phenotypes than male AD‐BXD mice. We found no relationship between motor function and decline in Ntg‐ and AD‐BXD strains. Finally, although there was a pronounced effect of age on grip strength, we did not observe an effect of transgene on this phenotype.

### 4.2 Influence of genetic background on sensorimotor traits and impact of the AD transgene on decline

We recently developed the first mouse model of genetic diversity in AD‐ the AD‐BXDs ‐ and showed that the inclusion of naturally occurring genetic variation introduces significant phenotypic variation in cognitive traits, including long‐term memory. Specifically, despite all strains carrying the same high‐risk 5XFAD transgene, some strains exhibit resilience to cognitive impairment while others show increased susceptibility.^19^ We now extend that analysis to sensorimotor traits, including coordination, balance, vestibular function and muscle strength. Similar to what we observed for cognitive abilities, there was a significant impact of genetic background on both baseline performance on motor tasks and both age‐ and AD‐related decline. As with long‐term memory, some strains exhibit resilience to age‐related and/or AD‐related impairments, exhibiting little decline with age, regardless of genotype. In contrast, some strains even showed improvement with age, suggesting modifier genes exist that influence both the normal motor function‐related aging process and AD‐related decline in motor abilities.

Humans exhibit substantial variation in longevity and healthspan,^30^, ^31^, ^32^ including the development of frailty and physical impairment in old age.^27^ Moreover, only a subset of AD patients exhibit motor‐related impairments.^4^, ^6^, ^33^, ^34^ The Ntg‐ and AD‐BXD panel exhibits similar phenotypic variation, suggesting that these panels represent a key resource for investigating the influence of genetic variation on age‐ and AD‐related frailty and motor decline. Heritability (*h* ^*2*^) estimates for all three sensorimotor domains evaluated in this study range from 0.6 to 0.9 (Table 1),^23^ suggesting a strong influence of genetic background on these phenotypes. Thus, future studies will exploit the power of the AD‐BXD panel as a mapping population to identify potential candidate modifier genes, which may represent novel targets for addressing sensorimotor decline in both normal aging and AD.

### 4.3 Sex differences in AD‐related motor decline

Although overall we did not find a significant main effect of sex on either the narrow beam or inclined screen phenotypes, for both traits we did find significant interactions between sex and age or genotype (Figures 1 and 4), with females performing worse than males in both instances. Thus, the mechanisms underlying both age‐related and AD‐related declines in vestibular function, balance and proprioception are exacerbated in females. This is consistent overall with reports that the incidence of AD is higher in women and that women are at higher risk of developing AD or a related dementia.^35^, ^36^, ^37^, ^38^ However, there are relatively few reports on motor phenotypes in AD and fewer still that stratify by sex, making comparisions between human studies and animal models difficult. In one study examining the relationship between gait speed decline and conversion to mild cognitive impairment (MCI) in men and women, women progress more rapidly to MCI once gait speed begins to decrease.^39^

### 4.4 AD‐related motor decline is likely distinct from both AD‐related cognitive decline and normal age‐related decline

Frailty and motor decline are common in aging humans,^27^ thus one possible explanation for the increased motor decline observed in both humans with AD and the AD‐BXDs is that the AD disease process acts to accelerate “normal” age‐related motor decline.^2^, ^3^ However, the lack of an observed correlation between two highly robust cognitive traits (CFA and CFM) and sensorimotor traits ‐ despite the high degree of heritability for all of these traits (Table 1)^19^ suggests that cognitive traits are controlled by distinct genetic mechanisms than the sensorimotor traits investigated here. In the two tasks that exhibited sensitivity to the AD transgene, narrow beam and incline screen, we found no correlation between the performance of the Ntg strains compared with the AD‐BXD strains, suggesting that the underlying genetic mechanisms regulating motor impairment in these two models is distinct. There is similarly no correlation between sensorimotor and cognitive performance across the AD‐BXD panel, including the Ntg‐BXD strains,^19^ suggesting that AD‐related motor impairment is distinct from both AD and age‐related cognitive decline. Thus, potential therapeutics targeted at memory loss and cognitive decline are unlikely to be effective at treating motor‐related deficits and may not forestall the loss of independence and need for long‐term care in patients, emphasizing the urgent need to better understand the impact of AD on noncognitive domains.

### 4.5 Limitations of this study

Notably, although grip strength has been reported to be associated with AD risk in humans,^2^, ^3^ we did not detect an effect of AD genotype on grip strength in the AD‐BXD panel at either age tested, although there was a pronounced effect of age on muscle strength in both Ntg‐ and AD‐BXD mice. However, there are several reports in human cohorts that indicate that lower extremity dysfunction is more closely associated with mild cognitive impairment (MCI) and AD than upper limb function.^33^, ^40^, ^41^, ^42^, ^43^ Our assessment of grip strength measured force exerted by all four limbs in each mouse and thus cannot distinguish between differential impairments between the forelimbs and hindlimbs in mice, although the narrow beam and inclined screen tasks would likely be sensitive to limb‐specific decline. Additionally, it is important to note that expression of the 5XFAD transgene used to generate the AD‐BXD reference panel is driven by a *Thy‐1* promoter and likely does not fully recapitulate the endogenous pattern or amyloid pathology or presenilin expression in the periphery. This limitation can be overcome by the use of a “knockin” model in which human mutations in *App* and *Psen1* are controlled by the endogenous promoters for both genes to produce a more translationally‐relevant organism‐wide expression pattern in both the CNS and peripheral tissues.

In human AD patients, motor impairment has been reported to occur prior to the onset of cognitive symptoms,^2^, ^3^, ^6^, ^33^ but in the AD‐BXD panel we did not observe significant impairment on any of the sensorimotor tasks used in this study at the earliest age measured (6 m). While we cannot exclude the possibility that motor decline does not precede cognitive deficits in the AD‐BXDs, a limitation of our study design is that motor function was measured at only two ages, 6 and 14 m. The average age at onset for cognitive impairment in the AD‐BXD panel is ~10 m,^19^ thus motor performance at 6 m of age may simply be too early of an age to detect a preclinical decline in sensorimotor function in the AD‐BXDs. In support of this hypothesis, although not significant (*P* > .05), we do see a trend for worse narrow beam performance in the AD‐BXDs compared with Ntg‐BXDs at 6 m of age (Figure 1B).

Some caution is warranted in the interpretation of narrow beam data when considering the behavior of mice during this task. We report strains with averages of latency >120 seconds (Figure 1C), suggesting that some strains have a large number of animals that simply did not move while on the beam or failed to remain on the beam. As a result, in some cases, an increase in time to cross the beam may not necessarily be due to an impairment of sensorimotor performance, but rather another behavioral output such as anxiety that influences performance on these tasks.

Genetic reference panels such as the BXD family used here represent powerful tools for the analysis and genetic mapping of quantitative traits such as the sensorimotor traits investigated here. As such, they are ideal for discovering new genetic loci associated with complex traits.^44^ The high degree of heritability for the traits we examined here ($h_{\mathrm{RI}\bar{x}}^{2}$ ≈ 0.6‐0.9) suggests they are amenable to QTL mapping to identify candidate genes and loci associated with sensorimotor performance. However, the number of strains used in the present study is insufficient for this approach and additional strains will be needed to ensure we are adequately powered for such analysis. That said, we are still able to investigate the relative contributions of the two parental genomes of the BXD family (C57Bl/6J and DBA/2J) and how their influence may differ between cognitive and noncognitive phenotypes. For example, we recently reported that the B6 genetic background confers resilience to the cognitive effects of the 5XFAD transgene.^19^ However, the B6 and D2 genomes may influence motor‐related phenotypes differently from cognitive phenotypes, as evidenced by the lack of correlation between contextual fear memory and acquisition and the narrow beam and inclined screen tasks (Figures 3 and 5).

### 4.6 Conclusions

The results presented here provide further evidence for the translational utility of our novel AD‐BXD panel, which we now show also exhibits sensorimotor decline similar to that reported in human AD patients. Moreover, this decline is strongly modified by genetic background and exhibits a high degree of heritability, consistent with the human disease. Thus, the AD‐BXD panel represents a novel tool to investigate sensorimotor deficits in normal aging and AD and facilitate the discovery of novel targets to address these deficits. Our finding that there is no correlation between motor and cognitive phenotypes in either the AD strains or the normal aging controls indicates that motor decline likely occurs via a mechanism distinct from cognitive decline in both AD and normal aging individuals. Future work will incorporate additional strains for genome‐wide mapping to identify potential modifier genes and omics analysis of CNS regions associated with sensorimotor tasks.

## AUTHOR CONTRIBUTIONS

K.M.S.O., S.M.N. and C.C.K. conceived of the experiments. S.M.N. conducted the behavioral experiments. K.M.S.O., A.R.O and C.C.K. designed the analyses. A.R.O. and K.M.S.O. performed the data analysis. A.R.O., C.C.K., S.M.N., A.R.D and K.M.S.O. contributed to the interpretation of results. K.M.S.O. wrote the manuscript. All authors approved of the final manuscript.
